# Identification of a Prognostic Risk Signature of Kidney Renal Clear Cell Carcinoma Based on Regulating the Immune Response Pathway Exploration

**DOI:** 10.1155/2020/6657013

**Published:** 2020-12-30

**Authors:** Guangzhen Wu, Yingkun Xu, Chenglin Han, Zilong Wang, Jiayi Li, Qifei Wang, Xiangyu Che

**Affiliations:** ^1^Department of Urology, The First Affiliated Hospital of Dalian Medical University, Dalian, China; ^2^Department of Urology, Shandong Provincial Hospital, Cheeloo College of Medicine, Shandong University, Jinan, China; ^3^School of Business, Hanyang University, Seoul, Republic of Korea

## Abstract

**Purpose:**

To construct a survival model for predicting the prognosis of patients with kidney renal clear cell carcinoma (KIRC) based on gene expression related to immune response regulation.

**Materials and Methods:**

KIRC mRNA sequencing data and patient clinical data were downloaded from the TCGA database. The pathways and genes involved in the regulation of the immune response were identified from the GSEA database. A single factor Cox analysis was used to determine the association of mRNA in relation to patient prognosis (*P* < 0.05). The prognostic risk model was further established using the LASSO regression curve. The survival prognosis model was constructed, and the sensitivity and specificity of the model were evaluated using the ROC curve.

**Results:**

Compared with normal kidney tissues, there were 28 dysregulated mRNA expressions in KIRC tissues (*P* < 0.05). Univariate Cox regression analysis revealed that 12 mRNAs were related to the prognosis of patients with renal cell carcinoma. The LASSO regression curve drew a risk signature consisting of six genes: TRAF6, FYN, IKBKG, LAT2, C2, IL4, EREG, TRAF2, and IL12A. The five-year ROC area analysis (AUC) showed that the model has good sensitivity and specificity (AUC >0.712).

**Conclusion:**

We constructed a risk prediction model based on the regulated immune response-related genes, which can effectively predict the survival of patients with KIRC.

## 1. Introduction

Worldwide, renal cell carcinoma (RCC) is a common urinary system tumor, accounting for approximately 90% of all malignant renal tumors. Kidney renal clear cell carcinoma (KIRC) is the most common pathological subtype, accounting for approximately 75% of all RCCs [1]. In 2018, approximately 65,340 patients with kidney cancer were diagnosed in the United States alone, and nearly, 14,970 patients died from kidney cancer [2]. The latest statistics show that the incidence of KIRC is increasing at an annual rate of 2% [3]. Presently, patients with early KIRC mainly rely on surgical treatment. In the early stages of the disease, most patients do not present specific symptoms; thus, approximately one-third of patients have metastatic disease at the time of diagnosis. Patients with metastases and relapses lose the opportunity for radical surgery and instead undergo traditional radiotherapy and chemotherapy [4]. Although some molecularly targeted drugs for KIRC have been used, most patients eventually develop drug resistance [5]. Therefore, mining new target biomarkers related to KIRC diagnosis and treatment is a hot topic in current cancer research and an urgent task.

In recent years, the relationship of the immune system in the development and progression of cancer has become critical for research. The interaction between the immune system and the tumor can be divided into three stages: immune elimination, immune hold-up, and immune escape [6]. In the immune elimination stage, the body endogenously suppresses tumor cells through gene repair, aging, and apoptosis. Simultaneously, the danger signals produced by apoptotic cells and the antigens produced by tumor cells stimulate the immune system; thus, innate and adaptive immunity work together to create an antitumor effect. After the immune elimination phase, the remaining mutating tumor cells interact with the adaptive immune cells and pass through the immune phase. At this time, the growth of tumor cells is prevented by immune mechanisms. When the balance between tumor cells and the immune system is disrupted, however, tumor cells generate immune escape environments by producing immunosuppressive cytokines, such as VEGF and TGF-*β*, and recruiting regulatory immune cells with immunosuppressive effects, such as TREG and MDSC [7]. As one of the biological characteristics of tumors, the immune escape phase shows the dual role of the immune system in tumor occurrence and development.

While the tumor-infiltrating immune cells can effectively control tumor cell occurrence and development and eventually eliminate tumor cells, they can also help the tumor avoid immune attack and promote tumor growth [8, 9]. In addition, in the process of tumor generation, exogenous pathways mediated by innate immune cells and endogenous pathways mediated by tumor cells work together to produce tumor-promoting inflammation in the tumor microenvironment, prompting tumors to break through the immune defense line [10]. Thus, tumor immunotherapy has become an important approach to tumor treatment.

In this study, we focused mainly on the potential role of genes related to immune response regulation in KIRC and successfully used these genes to establish a prognostic risk signature for patients with KIRC.

## 2. Materials and Methods

### 2.1. Data Collection

In June 2020, we downloaded the mRNA sequencing data of 539 cases of KIRC tissue and 72 cases of normal renal tissue from the TCGA database website with the clinicopathological data of patients (https://portal.gdc.cancer.gov/). The clinical information included age, sex, pathological stage, histological grade, survival time, and survival status. The pathway of immune response regulation was taken from the GSEA website (https://www.gsea-msigdb.org/gsea/index.jsp). We collected 33 genes related to this pathway for this study.

### 2.2. Construction of the PPI Network

The STRING database (http://www.string-db.org) collects and integrates known and predicted protein-protein association data for many organisms, including humans [11]. In this study, the STRING database was used to predict the interaction between proteins encoded by the related genes regulating the immune response. The protein-protein interaction (PPI) was constructed on the condition that the interaction combination score was greater than 0.4. Unlinked protein molecules were removed, and the interaction data and corresponding images on the remaining protein molecules were arranged.

### 2.3. Data Processing and Analysis

Our data processing and statistics were implemented using multiple extension packages based on the *R* language. First, we drew a heat map reflecting the expression of genes that regulate the immune response in KIRC. The “Pheatmap” extension package was used to draw heat maps, while the “limma” extension package was used to analyze mRNA differences. Then, we conducted a single factor COX analysis of these molecules in KIRC to show the relationship between these molecules and the prognosis of patients with kidney cancer. Next, we used the “corrplot” extension software package to map the coexpression relationship between genes involved in regulating the immune response. The “glmnet” and “survival” extension software packages were used to draw the LASSO regression curve and survival curve. The risk score was calculated based on a linear combination of the Cox coefficient and gene expression. The following calculation formula was used for the analysis: risk score = Σ^*N*^_i=1_ (Expi^*∗*^Coei). *N*, Coei, and Expi represent the gene number, coefficient value, and level of gene expression, respectively. The median was used to divide the patients into low-risk and high-risk groups. The corresponding survival curve was drawn. To verify the accuracy of the model, we used the “survival ROC” expansion package to obtain five-year and ten-year ROC curves. Finally, based on this model, we analyzed the correlation with the pathological features of RCC seen in patients and displayed the correlation as a heat map.

### 2.4. TIMER Database Analysis

TIMER is a database for the systematic analysis of various types of tumor immune information (https://cistrome.shinyapps.io/timer/) [12, 13]. The TIMER database contains data from 10897 samples of 32 cancers from TCGA and can assess the abundance of tumor immune invasion. The “Gene” module was used to analyze the correlation between the expression level of the target gene in KIRC and the infiltration abundance of immune cells. In the TIMER database, we analyzed the correlation between the genes that construct risk signatures and tumor immune infiltrating cells and used the Spearman correlation coefficient to express their relationship.

## 3. Results

### 3.1. The Expression of Regulated Immune Response-Related Genes in KIRC and the Interaction between Various Molecules

**Figure 1:**
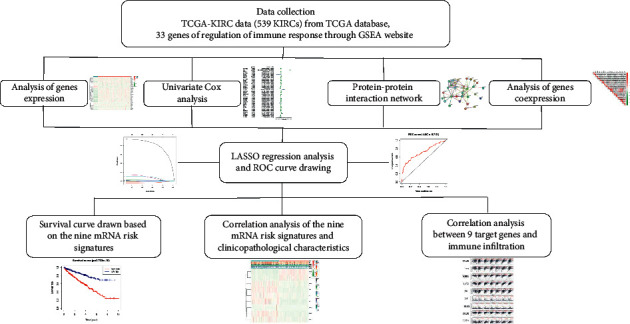
The flow chart of the study design and analysis.

**Figure 2:**
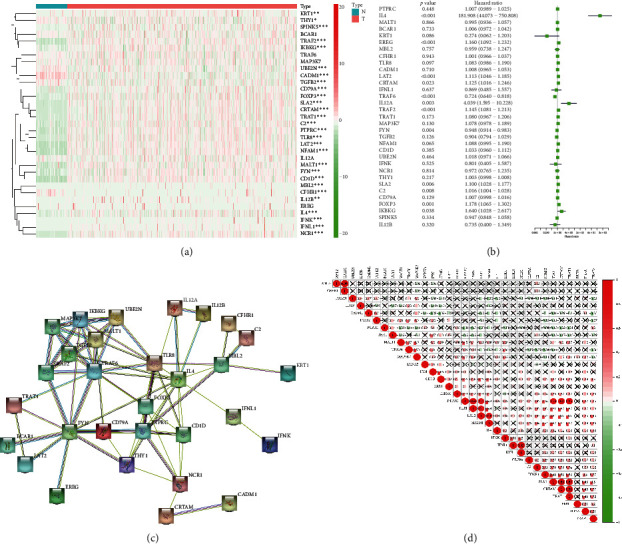
(a) A heat map showing the expression of genes that regulate the immune response between renal clear cell carcinoma and normal tissues. (b) A heat map showing the hazard ratio analysis of genes related to immune response regulation in KIRC. (c) The PPI network between immune response-related molecules. (d) Coexpression between immune-related molecules. ^*∗*^*P* < 0.05; ^*∗∗*^*P* < 0.01; ^*∗∗∗*^*P* < 0.001.

A flow chart displaying the overall design and analysis process of this research is presented in Figure 1. To explore the expression of genes related to the immune response regulation in clear cell RCC, we combined the *R* language with the mRNA expression data from the KIRC dataset in the TCGA database to draw a heat map of these genes. Twenty-eight of 33 genes were differentially expressed in KIRC tissues and normal kidney tissues (Figure 2(a)). Thus, the pathway regulating the immune response plays an important role in the development of clear cell RCC. The univariate COX analysis revealed ten molecules, IL4, EREG, LAT2, CRTAM, IL12A, TRAF2, SLA2, C2, FOXP3, and IKBKG, that are risk factors for the occurrence and development of KIRC. TRAF6 and FYN play a protective role in the occurrence and development of KIRC (Figure 2(b)). The interactions between various molecules are displayed in the PPI network (Figure 2(c)). The subsequent coexpression analysis revealed a strong coexpression relationship between MBL2 and CFHR1 (Figure 2(d)).

### 3.2. Construction and Verification of Risk Signatures

**Figure 3:**
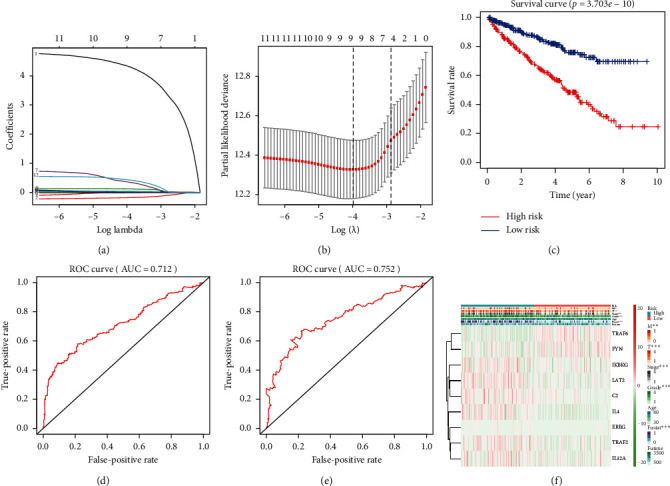
(a)-(b) Establishment of a risk signature using the LASSO regression curve and verification. (c) The survival curve analysis in KIRC based on the risk model. (d) Five-year ROC curve. (e) Ten-year ROC curve. (f) The relationship with clinicopathological features based on this model. ^*∗∗*^*P* < 0.01; ^*∗∗∗*^*P* < 0.001.

To establish risk signatures in KIRC using genes related to the regulation of the immune response, we used lasso regression analysis to select a model consisting of nine genes (Figure 3(a)) and cross-validated the feasibility of this model (Figure 3(b)). Using this model, patients with KIRC can be divided into high-risk and low-risk groups. The overall survival rate of patients in the high-risk group was significantly lower than that of patients in the low-risk group (*P*=3.703*e* − 10) (Figure 3(c)). R3.6.0 software was used to draw the ROC curve of the model to evaluate the model's sensitivity and specificity. The model predicts the area under the ROC curve (AUC) of the patients' 5-year survival rate (Figure 3(d)) and 10-year survival rate (Figure 3(e)). These rates were 0.712 and 0.752, respectively, indicating that the model has good sensitivity and specificity. These results suggest that the prognostic model we constructed can predict the survival of patients with clear cell RCC. When we combined this model with clinicopathological features, we found that this model was correlated with *M*, *T*, stage, grade, and fustat parameters (Figure 3(f)).

### 3.3. The Relationship between Regulated Immune Response-Related Genes and Various Immune Cells

**Figure 4:**
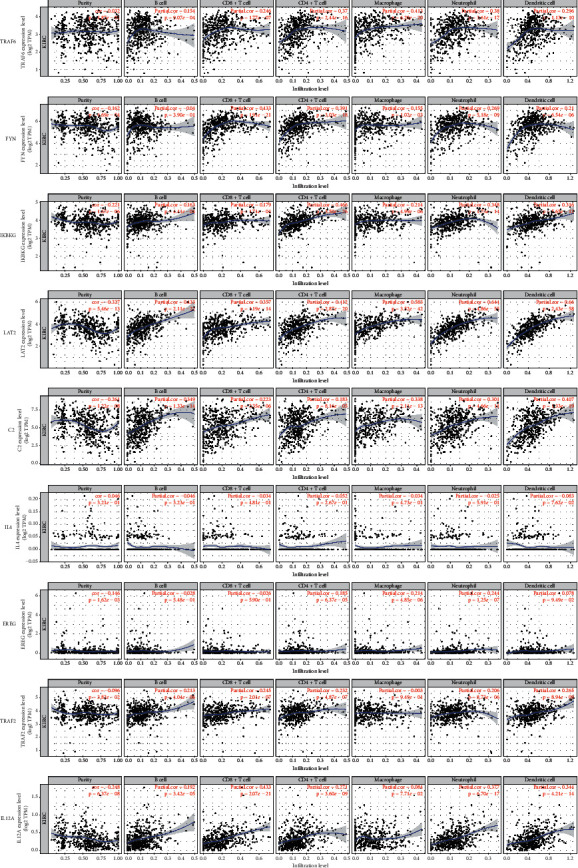
Correlation between TRAF6, FYN, IKBKG, LAT2, C2, IL4, EREG, TRAF2, IL12A, and various immune cells.

The results showed that there were correlations between the expressions of IKBKG, LAT2, and C2 and the infiltration of purity, B cells, CD8+ T cells, CD4+ T cells, macrophages, neutrophils, and dendritic cells. In addition, there was a correlation between the expressions of TRAF6 and IL12A and the infiltration of B cells, CD8+ T cells, CD4+ T cells, macrophages, neutrophils, and dendritic cells. There was a correlation between the expression of FYN molecules and the infiltration of purity, CD8+ T cells, CD4+ T cells, macrophages, neutrophils, and dendritic cells. Furthermore, there was a correlation between the expression of TRAF2 and the infiltration of B cells, CD8+ T cells, CD4+ T cells, neutrophils, and dendritic cells. There was a correlation between EREG expression and the infiltration of purity, CD4+ T cells, macrophages, and neutrophils (Figure 4).

## 4. Discussion

Bioinformatics analysis methods have been increasingly used in the medical field [14, 15]. In this study, we used various bioinformatics methods to deeply study the potential role of pathways regulating immune responses in KIRC. The KIRC prognostic model illustrates the relationship between the pathway regulating immune responses and the occurrence and development of KIRC. The prognostic model can effectively predict the overall survival rate of patients with KIRC. The Timer Online website suggests that these nine target genes are related to the infiltration of different immune cell types. This discovery provides a new strategy for the study of KIRC prognosis from the perspective of immune cell infiltration.

TRAF6, from the TRAF family, is involved in the transduction of inflammatory signals [16, 17]. Studies have shown that it can regulate tumorigenesis and angiogenesis in lung and pancreatic cancers [18–20]. In addition, it has been reported that TRAF6 inhibits the transfer of colorectal cancer by regulating autophagy [21]. Chen et al. confirmed that the activation of the FYN protein promotes the phosphorylation of Sam68, protecting pancreatic cancer cells from apoptosis and promoting the proliferation and metastasis of pancreatic cancer cells [22]. The FYN protein plays a role in promoting cancer by regulating the translation and activity of COX2 in prostate cancer [23]. In addition, FYN protein induces EMT in breast cancer, promoting tumor cell invasion and metastasis [24]. The FYN protein is also associated with breast cancer chemotherapy drug sensitivity [25]. A previous study showed that IKBKG in KIRC could prevent cancer cells from entering apoptosis by regulating HIF, promoting cancer cell survival and tumor metastasis [26]. LAT2 is overexpressed and plays a carcinogenic role in pancreatic cancer. It can also reduce the sensitivity of gemcitabine by modulating the LAT2-mTOR-LDHB signaling pathway [27]. Existing research shows that C2 gene polymorphism is closely related to chronic hepatitis B, hepatitis B virus-related cirrhosis, and liver cancer [28]. Chang et al. found that the high IL4 expression in KIRC is associated with increased tumor recurrence and decreased patient survival. In KIRC, the parameter IL4/IL13 is an independent prognostic factor for RFS and OS [29, 30]. EREG plays a carcinogenic role by binding to EGFR in various human tumors, such as colorectal cancer [31, 32], nonsmall cell lung cancer [33–35], and gliomas [36]. TRAF2 is similar to TRAF6 and plays an important role in inflammation, immune response, and malignant tumor infiltration [37–40]. Similarly, our results show that both TRAF2 and TRAF6 are significantly related to B cells, CD8+ T cells, CD4+ T cells, neutrophils, and dendritic cells. This shows that there is a strong correlation between TRAF2 and TRAF6 and immune infiltration. In addition, TRAF2 is upregulated in breast cancer, pancreatic cancer, and other malignant tumors [41, 42] and is significantly related to the prognosis of patients with prostate cancer [43]. Zhang et al. found that IL12A showed significantly high expression in differentiated thyroid cancer, was associated with disease invasiveness, and was an independent predictor of the prognosis of differentiated thyroid cancer [44].

The above indicates that these target genes utilize different molecular mechanisms in participating in the occurrence and development of various human malignant tumors. These target genes can promote malignant transformation in some tissues and inhibit tumorigenesis in other tissues. The abnormal expression of these molecules is closely related to the clinicopathological characteristics of the tumor and the prognosis of patients. In the future, these genes can become potential biological targets for tumor prevention, diagnosis, and treatment. Among them, studies on the potential functions of TRAF6, FYN, LAT2, and other molecules in KIRC are still relatively few. Therefore, the specific molecular mechanism of how these molecules affect tumorigenesis and development in the future is worthy of in-depth study.

Although this study has made progress in the prognosis of KIRC, there are some limitations. The study used public datasets, making it impossible to obtain all the information needed by each patient. This entails that some patients with acute infections, diseases of the immune system, or using anti-inflammatory drugs may have been included in this study. Ideally, these patients should be excluded. In addition, the gene expression data of normal kidney tissue are less than that of tumor tissue in the currently available tumor genome atlas database, which lead to an imbalance in the sample when collecting the data. However, with the development of current high-throughput gene expression detection technology, more and more gene expression data will be available for investigation. In future studies, we will collect more data on patients with KIRC and normal tissue data and continue to conduct further research.

## 5. Conclusion

In summary, we constructed a risk model for the survival of patients with clear cell renal cell carcinoma composed of 9 genes that modulate the immune response. The model has good sensitivity and specificity and indicates that patients in the high-risk group have a greater risk of death. The study showed that the establishment of multigene prognostic models can provide accurate prognostic guidance and has an important reference value for the selection of individualized treatment plans. The risk model can effectively predict the survival of patients with permeable clear cell renal cell carcinoma. Even so, the model still needs to be further verified using large-scale multicenter evidence-based medical evidence.

## Data Availability

The data used to support the findings of this study are available from the corresponding author upon request.

## Abbreviations

KIRC: Kidney renal clear cell carcinoma
TCGA: The cancer genome atlas
GSEA: Gene set enrichment analysis
TRAF6: TNF receptor associated factor 6
FYN: FYN protooncogene
IKBKG: Inhibitor of nuclear factor kappa B kinase regulatory subunit gamma
LAT2: Linker for activation of T cells family member 2
C2: Complement C2
IL4: Interleukin 4
EREG: Epiregulin
TRAF2: TNF receptor associated factor 2
IL12A: Interleukin 12A
RCC: Renal cell carcinoma
TREG: Regulatory T cell
MDSC: Myeloid-derived suppressor cell.
